# Recurrent Early Pregnancy Loss and Congenital Thrombophilia: A Prospective Study

**DOI:** 10.3390/jcm13226871

**Published:** 2024-11-15

**Authors:** Asma Basha, Yasmine Alkhatib, Tamara Tashtoush, Maysa Yousef, Laila Oweidi, Mohammad Alkhatib, Sally Al-Aqrabawi, Yazun Jarrar, Abdalla Awidi

**Affiliations:** 1 Department of Obstetrics and Gynecology, School of Medicine, The University of Jordan, Amman 11942, Jordan; a.basha@ju.edu.jo; 2 Department of Obstetrics and Gynecology, Jordan University Hospital, Amman 11942, Jordan; yas8170045@ju.edu.jo (Y.A.); tamara_tashtoush_90@hotmail.com (T.T.);; 3 Thrombosis Hemostasis Laboratory, School of Medicine, The University of Jordan, Amman 11942, Jordanm_alkhatib2004@yahoo.com (M.A.); 4 Cell Therapy Center, The University of Jordan, Amman 11942, Jordan; sally2ms@gmail.com; 5 Department of Basic Medical Science, Faculty of Medicine, Al-Balqa Applied University, Al-Salt 19117, Jordan; yazun.jarrar@bau.edu.jo

**Keywords:** recurrent pregnancy loss, thrombophilia, PT, PTT, FXIII, MTHFR, PAI-1, EPCR

## Abstract

**Background/Objectives**: This study aims to investigate the role of congenital single nucleotide thrombophilia in young females with early recurrent pregnancy loss (RPL). **Methods**: We studied 120 pregnant females with RPL and 80 matched females as a control with no RPL. Females were aged ≤ 35 years, had at least two consecutive first-trimester RPLs, and the acquired cause of RPL was excluded. A matched control group of 80 pregnant women with no RPL was studied. Coagulation tests included prothrombin time (PT), partial thromboplastin time (PTT), thrombin time (TT), a Factor XIII functional assay, and detecting IgM and IgG anti-beta2-Glycoprotein I (β2GPI) antibodies by an ELISA. The DNA samples were tested for Factor V Leiden, Factor II G20210A, Methylenetetrahydrofolate reductase (MTHFR C677T, A1298C), FXIII V34L, plasminogen activator inhibitor-1 (PAI-1) 4G/5G, endothelial protein C receptor (EPCR) A4600G, and endothelial protein C receptor (EPCR) G4678C. **Results**: Of the single nucleotide gene mutations investigated, the most relevant mutations were MTHFR C677T, MTHFR A1298C, heterozygous FXIII Val34Leu, and heterozygous FXIII 1694 C>T. Each of them conferred a statistically significant effect. There was a statistically significant protective role for the endothelial protein C receptor (EPCR) A2/A2, wild FXIII Val34Leu, and heterozygousFXIII1694 C>T. **Conclusions**: Our findings suggest the important role of congenital single nucleotide thrombophilia mutations in young Middle Eastern women with early RPL, particularly MTHFR mutations and FXIII Val34Leu. We found a protective effect of EPCR A2/A2, wild FXIIIVal34Leu, and heterozygous FXIII1694 C>T. We recommend additional studies to explore detrimental factors and protective factors.

## 1. Introduction

Recurrent pregnancy loss (RPL), defined as the loss of three or more pregnancies before 20 weeks of gestation, affects approximately 0.7% of the population (0.5–0.8%) [1,2,3]. While relatively rare, RPL carries significant emotional and psychological burdens for affected individuals and their families. The underlying causes of RPL are multifactorial, with a range of risk factors identified, including a maternal age below 20 or above 35, an abnormal body mass index (either low or high), being of Black ethnicity, advanced paternal age (above 40), and lifestyle factors such as smoking and alcohol consumption [1]. Despite advances in reproductive medicine, a clear and universally accepted definition of RPL remains elusive. While the European Society for Human Reproduction and Embryology (ESHRE) and the World Health Organization (WHO) define RPL as three or more consecutive miscarriages before 20 weeks of gestation [2,4], recent research has called for a revision, suggesting that two consecutive miscarriages may also constitute RPL [4]. This ongoing debate emphasizes the need for a better understanding of the condition’s underlying causes to facilitate earlier diagnosis and treatment.

One area of growing interest in RPL research is the role of congenital thrombophilia. Thrombophilia, a predisposition to abnormal blood clot formation due to inherited or acquired abnormalities in the coagulation system, has been linked to adverse pregnancy outcomes, including RPL [3]. However, the relationship between congenital thrombophilia and RPL remains controversial. Previous studies have produced conflicting findings, with some indicating an increased risk of RPL in women carrying specific thrombophilia mutations such as Factor V Leiden and Methylenetetrahydrofolate reductase (MTHFR), particularly in those with homozygous or compound heterozygous mutations [5,6,7,8,9]. Inherited thrombophilia induces hypercoagulation that impairs the placental function with either arterial or venous thrombosis at the maternal–fetal interface. This mechanism forms the likely basis of RPL [10]. Conversely, other investigations have failed to demonstrate any significant association between inherited thrombophilia and recurrent miscarriage [11,12]. These discrepancies may arise from differences in study design, sample size, or the specific thrombophilia mutations investigated, and further research is needed to clarify the potential role of thrombophilia in RPL.

In this prospective controlled study, we aimed at investigating the role of congenital thrombophilia factors in pregnant women who have experienced recurrent pregnancy loss at twelve weeks of gestation or earlier. Through a careful examination of thrombophilia markers and their potential impact on pregnancy outcomes, we sought to contribute to the ongoing dialog surrounding RPL and improve the understanding of its underlying etiologies. Our findings suggest the important role of congenital single nucleotide thrombophilia mutations in young Middle Eastern women with early RPL, particularly MTHFR mutations and FXIII Val34Leu. We found a protective effect of EPCR A2/A2, wild FXIIIVal34Leu, and heterozygous FXIII1694 C>T.

## 2. Materials and Methods

### 2.1. Study Design and Sample

This is a prospective work on pregnant women with recurrent early pregnancy loss (RPL). The work was approved by the Institutional Review Board (IRB) of the Cell Therapy Center, Amman, Jordan (CTC) [IRB-CTC/6-2018/03]. All research activities were conducted in accordance with the ethical standards laid down in the 1964 Declaration of Helsinki and its later amendments. Signed written informed consent was obtained from all participants. According to the records from Jordan University Hospital, an average of 150 Jordanian (Arabic ethnicity) pregnant women with RPL were diagnosed at the period of the study. A sample size of 105 was calculated to be necessary to represent the population of pregnant women with RPL, based on a power of test (1 − β) of 0.8, a 5% margin of error, and a 95% confidence level. For this work, we used a sample consisting of 120 female patients with RPL (*n* = 120).

The gestational age for each patient was calculated from the first day of the last menstrual period. The sample consisted of 120 female patients with RPL (n = 120) that received health care at JUH and met the following inclusion criteria: age ≤ 35 years, at least 2 consecutive first-trimester RPLs in less than 12 weeks, no hyperglycemia in pregnancy, no chronic medical illnesses, and no acquired cause of RPL. In addition, patients who did not fulfill the following exclusion criteria were excluded: age > 35 years, RPL is not consecutive, RPL is beyond the first trimester, acquired cause for RPL is known, or a medical illness that prevents the inclusion as outlined in the inclusion criteria. For the control group, 80 age-matched pregnant women were taken who had two consecutive pregnancies without miscarriage (n = 80) and met the rest of the inclusion criteria. Exclusion criteria were age >35 years, pregnancy loss at any age and any cause, and any significant medical illness. No patients with a history of pregnancy loss were included in the control group.

### 2.2. Biochemical Investigation

A blood sample from patients and controls was taken for the required coagulation and DNA tests for thrombophilia. Samples were obtained in EDTA, sodium citrate, and plain tubes. A total of 20 mL of blood was obtained. EDTA tubes were used to obtain DNA. Samples were stored at −80 °C until tested. Sodium citrate tubes were used for the clotting assays for prothrombin time (PT), partial thromboplastin time (PTT), and thrombin time (TT) were performed as per established methods [13,14].

Serum was obtained from the plain tube and kept in −80 °C until tested for antibodies using an enzyme immunoassay kit for the detection of IgM and IgG anti-β2GPI antibodies using ELISA™ and a 2GPI IgM and IgG Kit (Louisville APL Diagnostics, Texas City, TX, USA, TX 77590-7451) [15].

The quantitative functional FXIII activity was determined with a spectrophotometric ammonia release assay using a BIOPHEN Factor XIII kit (HYPHEN BioMed, Neuville-sur-Oise, France) according to the manufacturer’s instructions. Briefly, the polymerase chain reaction was used to amplify the F13A1 gene provided by HGNC, symbol: HGNC:3531, that encodes the coagulation Factor XIII A subunit; exon 12 was amplified with a total volume of 25 μL using 2X Pfu PCR Master Mix (Biotechrabbit, Berlin, Germany) under the following conditions: an initial step of 2 min at 95 °C, followed by 35 cycles at 95 for 1 min, 56 °C for 1 min, 72 °C for 1 min, and a final extension at 72 °C for 10 min. The primers were newly designed (IDT, Iowa, USA) and checked for specificity using the Primer Blast tool and Primer 3 software [16]. For gel electrophoresis, the amplified products were separated on 2% agarose gel in 1X TBE buffer to ensure the size and quality of the band, suggesting no contaminating or unspecific PCR products. A group of samples along with 100 pb ladder (Biotechrabbit, Berlin, Germany) PCR products were purified using the GenUP™ PCR Clean-up kit (Biotechrabbit, Berlin, Germany). For Sanger sequencing, the direct sequencing of the coding region for exon 12 was performed using a BigDye™ Terminator v3.1 Cycle Sequencing kit (applied biosystems, Thermo Fisher Scientific, Massachusetts, USA), and the fragments were gel-purified using NucleoSEQ ^®^ (MN, Düren, Germany); the capillary electrophoresis was performed using a 3500 Genetic Analyzer sequencer (Applied Biosystems, Foster City, CA, USA). The DNA samples were stored at −20 °C and were tested for Factor V G1691A (Leiden), Factor V H1299R, Factor II G20210A, Methylenetetrahydrofolate reductase (MTHFR C677T, A1298C), Factor XIII V34L, plasminogen activator inhibitor-1 (PAI-1) 4G/5G, endothelial protein C receptor (EPCR) A4600G, and endothelial protein C receptor (EPCR) G4678C. The test was performed using the CVD StripAssay^®^ T (Vienna Lab, Wien, Austria) as per the manufacturer’s instructions.

### 2.3. Statistical Analysis

The distribution of genotypes between the control and patient groups was assessed using the chi-square (χ2) test. Additionally, the genotypic frequency was scrutinized for adherence to the Hardy–Weinberg equilibrium using the χ2 test. Continuous data comparing variables between the control and patient groups were evaluated using the one-tailed Student’s t-test. The ANOVA one-way test was used to compare the results. All statistical analyses were conducted using SPSS software, version 26, by IBM, Armonk, NY, USA, with a significance threshold set at a *p*-value below 0.05.

## 3. Results

### 3.1. Demographic and Relevant Clinical Characteristics of the Patient vs. Control Groups

We first described the clinical characteristics of the patients’ samples (Table 1). The two groups (patients with RPL vs. controls) were well matched for both age and weight, ensuring comparability. A total of 80 control subjects and 120 patients (n = 120) were included in the analysis. The two groups were matched in terms of age and weight. The mean age was 32.26 ± 4.15 years for the control group and 30.95 ± 5.67 years for the patient group, with no statistically significant difference between them (*p* = 0.14). Similarly, the mean weight of the control group (74.64 ± 12 kg) and the patient group (75.65 ± 14.41 kg) was comparable, with no significant difference (*p*= 0.61). However, significant differences were observed in the reproductive outcomes between the two groups. The number of miscarriages was significantly higher in the patient group, with a mean of 3.38 ± 2.57 miscarriages, while the control group reported no miscarriages (*p* < 0.001). Additionally, the number of live births was significantly lower in the patient group, with a mean of 2.34 ± 1.34 live births compared to 3.5 ± 0.9 live births in the control group (*p* = 0.001). These findings indicate that while the control and patient groups were similar in terms of age and weight, there were significant differences in reproductive history, with the patient group experiencing a higher number of miscarriages, confirming the validity of our patient group.

### 3.2. Comparison of FofII Activity; B2GPI Antibodies (Ab2gpi); and IgG, IgM, and Bleeding Indices of the Patient vs. Control Groups

We next moved on to analyzing Factor XIII (FXIII) activity, β2GPI antibodies (IgM and IgG), and bleeding parameters including prothrombin time (PT), partial thromboplastin time (PTT), and thrombin time (TT) between the control and patient groups (Table 2). FXIII activity was similar between the two groups. The control group had an average FXIII activity of 65.01 ± 54.03, while the patient group had a slightly lower average FXIII activity of 55.44 ± 54.79, albeit not being statistically significant (*p* = 0.23). Next, we examined β2GPI antibody levels; patients exhibited higher mean IgM (4.51 ± 5.43) and IgG (5.23 ± 5.76) levels compared to controls (IgM: 3.60 ± 4.67; IgG: 3.79 ± 3.82). The difference in IgM levels was not statistically significant (*p* = 0.23); however, the difference in IgG levels approached statistical significance (*p* = 0.05). We moved on to measure coagulation parameters and found that there were no significant differences between the two groups. The mean PT was 13.69 ± 1.38 s for the control group and 13.75 ± 1.23 s for the patient group (*p* = 0.7). Similarly, PTT and TT values were comparable between the groups, with the control group having a PTT of 36.94 ± 1.55 s and a TT of 20.20 ± 1.22 s, while the patient group showed a PTT of 37.04 ± 1.58 s and a TT of 20.53 ± 1.44 s. The differences in PTT and TT were also not significant (*p* = 0.61 and *p* = 0.10, respectively). Collectively, these findings suggest no significant variations amongst the measured parameters with the exception of elevated β2GPI IgG levels in the patient group that was almost statistically significant.

### 3.3. Comparison of Genotype Frequencies Between Control and Patient Subjects

We next compared the distribution of genotypes for four relevant genetic polymorphisms between the control and patient groups. The relevance of comparing the distribution of genotypes for key genetic polymorphisms between control and patient groups is connected to understanding the potential genetic predispositions to RPL. Genetic polymorphisms such as FV G1691A (Leiden), FV His1299Arg, FII G20210A, and MTHFR C677T have been previously implicated in thrombophilia [17,18,19,20]. Exploring these polymorphisms in both control and RPL patient groups can help clarify their role in the pathology of RPL and reveal specific genetic patterns that may contribute to the increased risk. Our data suggest several trends in genotype frequencies for FV G1691A (Leiden, The Netherlands), FV His1299Arg, FII G20210A, and MTHFR C677T (Table 3).

#### 3.3.1. FV G1691A (Leiden)

The prevalence of the wild-type allele was higher in both groups (controls: 80%; patients: 86.7%), although it did not reach statistical significance (*p* = 0.26). Heterozygous genotypes were present in 16.3% of controls and 12.5% of patients, again showing no significant difference (*p* = 0.55). Homozygous individuals were rare in both groups, representing only 3.8% of controls and 0.8% of patients (*p* = 0.25).

#### 3.3.2. FV His1299Arg

The wild-type genotype for FV His1299Arg was similarly frequent in both groups (controls: 81.3%; patients: 78.3%; *p* = 0.61). Heterozygous individuals represented 16.3% of controls and 20% of patients, while homozygous individuals were rare in both groups (2.5% in controls and 1.7% in patients). Neither comparison reached statistical significance (heterozygous: *p* = 0.48; homozygous: *p* = 0.69).

#### 3.3.3. FII G20210A

A similar trend was seen for the FII G20210A polymorphism, with the wild-type genotype being dominant, yet again with no documented statistical significance (controls: 93.8%; patients: 96.7%; *p* = 0.30). Heterozygous individuals made up 6.3% of controls and 3.3% of patients (*p* = 0.30). Homozygous individuals were absent from both groups.

#### 3.3.4. MTHFR C677T

The MTHFR C677T polymorphism exhibited a notable difference between groups, as the frequency of the wild-type genotype was higher in the control group (60%) compared to the patient group (49.2%) (*p* = 0.12). Heterozygous individuals represented 37.5% of controls and 40.8% of patients (*p*= 0.67). Homozygous individuals for the MTHFR C677T polymorphism, however, showed a statistically significant difference (*p* = 0.04). Only 2.5% of controls were homozygous compared to 10% of the patient group. The odds ratio for homozygosity in the patient group was four (95% CI: 0.87 to 18.35), suggesting a potential association between the homozygous MTHFR C677T genotype and recurrent pregnancy loss (RPL).

#### 3.3.5. PAI-1 4G/5G Polymorphism

The distribution of the PAI-1 4G/5G polymorphism did not significantly vary between RPL cases and controls. The homozygous 4G/4G genotype was observed in 18.8% of the patients compared to 15.8% of controls (OR = 0.58). The heterozygous 4G/5G genotype appeared in 47.5% of the cases and 49.2% of controls (OR = 0.89). The homozygous 5G/5G genotype was present in 33.8% of cases and 35.0% of controls (OR = 0.88). These findings indicate a lack of significant association between the PAI-1 4G/5G polymorphism and RPL.

#### 3.3.6. EPCR Haplotypes (A1, A2, A3) and A4600G (G4678C)

An analysis of the EPCR haplotypes (A1, A2, A3) revealed a significant difference between cases and controls in the A2/A2 haplotype group. A2/A2 was observed in 17.5% of RPL cases compared to 5% (n = 6) of controls (OR = 0.006; 95% CI: 0.27–0.94), suggesting a possible protective effect against RPL, at least in this patient subpopulation. A1/A1, A1/A2, A1/A3, and A3/A3 showed no significant differences between patients and controls.

#### 3.3.7. FXIII Val34Leu Variant

A significant association was observed with the FXIII Val34Leu variant. The heterozygous genotype was more prevalent in RPL cases (11.3%, n = 9) compared to controls (24%, n = 19), showing an OR of 0.02 (95% CI: 0.96–4.77). Additionally, the wild-type (Val/Val) genotype was found in 87.5% (n = 70) of cases compared to 75.8% (n = 59) of controls (OR = 0.001). These results suggest that the Val34Leu variant, particularly in the heterozygous state, might be associated with an increased risk of RPL.

#### 3.3.8. FXIII c.1694 C>T (p. Pro565Leu)

The FXIII c.1694 C>T (Pro565Leu) variant showed a significant association with RPL. The heterozygous state was observed in 22.5% (n = 18) of cases compared to 11% of controls, with an OR of 0.03 (95% CI: 0.22–1.03). The wild-type (C/C) genotype was found in 73.8% of cases and 89% of controls (OR = 0.01). Interestingly, the homozygous T/T genotype was noted in 3.8% of cases but was absent in the control group.

#### 3.3.9. FXIII c.1704 A>G (p. Glu568=)

The FXIII c.1704 A>G (p. Glu568=) variant was present predominantly in the wild-type state, with 92.5% of cases and 91.5% of controls showing the wild-type genotype (OR = 0.87). The heterozygous A/G variant was observed in 5.3% (n = 4) of cases and 8.5% (n = 10) of controls (OR = 0.19). Only one homozygous G/G genotype was observed among cases and none in controls, with no statistically significant association between this variant and RPL (OR = 0.64).

The statistically significant findings are presented in Table 3 and further summarized in Table 4. Notably, the following mutations were more prevalent in the patient group than in the control group: homozygous MTHFR C677T (*p* < 0.04), homozygous MTHFR A1298C (*p* < 0.03), heterozygous FXIII Val34Leu (*p* < 0.02), and wild-type FXIII c.1694 C>T p. Pro565Leu (*p* < 0.01). These variants were associated with detrimental effects on pregnancy outcomes. Conversely, certain genetic findings appeared to confer a statistically significant protective effect. These included EPCR haplotype A2/A2 (*p* < 0.006), wild-type FXIII Val34Leu (*p*< 0.001), heterozygous FXIII c.1694 C>T p. Pro565Leu (*p* < 0.03), and homozygous FXIII c.1694 C>T p. Pro565Leu (*p* < 0.03).

## 4. Discussion

The role of congenital thrombophilia in recurrent pregnancy loss (RPL) remains contentious, with numerous studies yielding conflicting results due to variations in study design, patient demographics, and genetic testing methodologies [5,6,13]. Previous research has frequently implicated Factor V Leiden and the prothrombin gene mutation (PGM) as significant contributors to RPL risk [17,18,19,20,21,22]. Additionally, the MTHFR C677T mutation has been linked to an increased likelihood of recurrent miscarriages [23,24]. Another variant, FXIIIA Val34Leu, has been associated with a heightened risk of venous thrombosis and RPL [25].

However, our findings diverge from much of this earlier research. In our study population, there was no observed association between recurrent RPL and Factor V Leiden, FV His1299Arg, FII G20210A, PAI-1 4G/5G phenotypes, Factor XIII activity, or abnormalities in PT, PTT, or TT coagulation tests. Instead, we identified statistically significant associations between RPL and the following genetic mutations: homozygous MTHFR C677T (*p* < 0.04), homozygous MTHFR A1298C (*p* < 0.03), heterozygous FXIII Val34Leu (*p* < 0.02), and wild-type FXIII c.1694 C>T p. Pro565Leu (*p* < 0.01). These findings stand in contrast to earlier studies that highlighted associations with FV Leiden and FII mutations [25,26,27], suggesting that the discrepancies may stem from differences in patient populations, particularly in age, as our cohort may represent a younger demographic than those previously studied.

It is noteworthy that we were able to confirm a strong association between RPL and homozygous MTHFR C677T and A1298C mutations, as well as heterozygous FXIII Val34Leu. Additionally, we report a novel association between RPL and the wild-type FXIII c.1694 C>T genotype. Interestingly, we also identified significant protective effects from EPCR haplotypes A2/A2, wild-type FXIII Val34Leu, and both heterozygous and homozygous FXIII c.1694 C>T genotypes (*p* < 0.03). In a prospective cohort study of 7343 pregnant women to investigate the association of thrombophilia with placenta-mediated pregnancy complications, there were 507 (6.9%) women with FVL and or prothrombin gene mutations (PGMs); FVL and PGMs were associated with a relative risk of 1.04 (95% CI, 0.81–1.33) [17,18]. An extensive review has been published detailing many previous studies on the topic [19].

The management of RPL in women with inherited thrombophilia remains highly debated. Some experts advocate for prophylactic anticoagulation therapy in these patients, citing potential benefits in reducing miscarriage risk, while others argue that the evidence remains insufficient to justify such an approach, particularly given the increased risk of bleeding complications associated with anticoagulants [28,29]. As a result, individualized counseling and management tailored to the specific genetic and clinical profiles of these patients are essential.

Although this study offers meaningful findings, it is important to acknowledge its limitations. The relatively small sample size reduces the generalizability of the results, potentially limiting the study’s statistical power. Additionally, focusing exclusively on patients in their first trimester with two or more RPLs narrows the scope of applicability. Furthermore, the uniformity of the ethnic group, while providing some control over confounding variables, may restrict the broader relevance of the findings to more diverse populations. Lastly, if data collection occurred within less than three months post-miscarriage, it could have impacted the results. This timing can potentially affect variables related to RPL. A clearer specification of the timing of participants’ most recent miscarriage would indeed help improve the study’s interpretability and generalizability. These factors should be considered when interpreting the results and applying them to wider clinical settings.

Despite these limitations, our study contributes to the current body of knowledge by emphasizing the importance of early screening for single nucleotide thrombophilia gene mutations in young women experiencing recurrent pregnancy loss in the first trimester. This highlights a critical area that warrants further investigation to better understand the role of thrombophilia in pregnancy outcomes and guide future clinical practices.

The identification of inherited thrombophilia in women with RPL carries significant clinical implications. These patients may benefit from prophylactic anticoagulation during pregnancy; however, the optimal approach to thrombophilia management during pregnancy remains unresolved. The decision to use anticoagulants must be carefully balanced against the potential risks of bleeding and other adverse outcomes [28,29,30,31]. Further research is required to clarify the most effective treatment strategies for women with RPL associated with inherited thrombophilia, allowing for more informed personalized care.

## Figures and Tables

**Table 1:** Clinical parameters of control and patient subjects. * indicates statistical significance with *p*-value < 0.05.

Clinical Parameter	Controls	Patients	*p*-Value
Number of subjects	80 subjects	120 subjects	--
Age (mean ± SD)	32.26 ± 4.15 years	30.95 ± 5.67 years	0.14
Weight (mean ± SD)	74.64 ± 12 kg	75.65 ± 14.41 kg	0.61
Number of miscarriages (mean ± SD)	0	3.38 ± 2.57	<0.001 *
Number of alive babies (mean ± SD)	3.5 ± 0.9 babies	2.34 ± 1.34 babies	0.001 *

**Table 2:** Comparison of FXIII activity; B2GPI antibodies (aB2GPI); and IgG, IgM, and bleeding parameters between control and patient groups.

Subject Status		FXIII Activity	aB2GPI-M	aB2GPI-G	PT	PTT	TT
Control	Mean	65.01	3.60	3.79	13.69	36.94	20.20
	N	80	80	80	80	80	80
	SD	54.03	4.67	3.82	1.38	1.55	1.22
Patients	Mean	55.44	4.51	5.23	13.75	37.04	20.53
	N	120	120	120	120	120	120
	SD	54.79	5.43	5.76	1.23	1.58	1.44
	P	0.23	0.23	0.05	0.7	0.61	0.10

**Table 3:** Comparison of genotype frequencies between control and patient subjects.

		Subject Status		*p*-Value	Odds Ratio (95 CI)
		Controls	Patients		
FV G1691A (Leiden)	Wild	64 (80%)	104 (86.7%)	0.26	1.41 (95% CI: 0.63 to 3.15)
	Heterozygous	13 (16.3%)	15 (12.5%)	0.55	0.71 (95% CI: 0.32 to 1.59)
	Homozygous	3 (3.8%)	1 (0.8%)	0.25	0.21 (95% CI: 0.02 to 2.01)
FV His1299Arg	Wild	65 (81.3%)	94 (78.3%)	0.61	1.43; 95% CI: 0.83 to 2.45
	Heterozygous	13 (16.3%)	24 (20%)	0.48	0.39; 95% CI: 0.19 to 0.79
	Homozygous	2 (2.5%)	2 (1.7%)	0.69	0.02; 95% CI: 0.00 to 0.20
FII G20210A	Wild	75 (93.8%)	116 (96.7%)	0.30	6.83; 95% CI: 2.51 to 18.55
	Heterozygous	5 (6.3%)	4 (3.3%)	0.30	1.93; 95% CI: 0.51 to 7.41
MTHFR C677T	Wild	48 (60%)	59 (49.2%)	0.12	1.66; 95% CI: 0.89 to 3.09
	Heterozygous	30 (37.5%)	49 (40.8%)	0.67	0.84; 95% CI: 0.47 to 1.50
	Homozygous	2 (2.5%)	12 (10%)	0.04	4 (0.87 to 18.35)
MTHFR A1298C	Wild	45 (56.3%)	50 (41.7%)	0.45	1.70; 95% CI: 0.93 to 3.09
	Heterozygous	31 (38.8%)	54 (45%)	0.41	0.75; 95% CI: 0.41 to 1.38
	Homozygous	4 (5%)	16 (13.3%)	0.03	2.67 (0.86 to 8.26)
PAI-14G/5G	4G/4G	15 (18.8%)	19 (15.8%)	0.58	1.24; 95% CI: 0.63 to 2.46
	4G/5G	38 (47.5%)	59 (49.2%)	0.89	0.91; 95% CI: 0.53 to 1.55
	5G/5G	27 (33.8%)	42 (35%)	0.88	0.95; 95% CI: 0.53 to 1.71
EPCR Haplotypes (A1, A2 and A3) (A4600GG4678C)	A1/A1	11 (13.8%)	24 (20%)	0.28	0.63; 95% CI: 0.26 to 1.53
	A1/A2	30 (37.5%)	59 (49.2%)	0.12	0.31; 95% CI: 0.17 to 0.56
	A2/A2	28 (35%)	21 (17.5%)	0.006	0.5 (0.27 to 0.94)
	A1/A3	4 (5%)	7 (5.8%)	0.76	0.93; 95% CI: 0.30 to 2.89
	A2/A3	6 (7.5%)	7 (5.8%)	0.83	1.30; 95% CI: 0.50 to 3.41
	A3/A3	1 (1.3%)	2 (1.7%)	0.89	0.78; 95% CI: 0.09 to 6.79
FXIII Val34Leu	Wild	70 (87.5%)	91 (75.8%)	0.001	1.74; 95% CI: 1.06 to 2.85
	Heterozygous	9 (11.3%)	29 (24.2%)	0.02	2.1 (0.96 to 4.77)
FXIII c. 1694 C> T p. Pro565Leu	Wild	59 (73.8%)	105 * (89%)	0.01	2; 95% CI: 1 to 3
	Heterozygous	18 (22.5%)	13 (11%)	0.03	0.5 (0.22 to 1.03)
	Homozygous	3 (3.8%)	0 (0%)	0.03	0.09 (0.01 to 1.87)
FXIII c. 1704 A>G p. Glu568=	Wild	74 (92.5%)	108 * (91.5%)	0.87	1.29; 95% CI: 0.62 to 2.69
	Heterozygous	5 (6.3%)	10 (8.5%)	0.75	0.88; 95% CI: 0.36 to 2.13
	Homozygous	1 (1.3%)	0 (0%)	0.64	Not applicable

Abbreviations: FV: Factor V; FII: Factor II; MTHFR: Methylenetetrahydrofolate reductase; PAI-1: plasminogen activator inhibitor-1; EPCR: endothelial protein C receptor; FXIII: Factor XIII; CI: confidence interval. One control was homozygous for FXIII Val34Leu. * Two patients were not tested for FXIII codon 1694 C>T (p. Pro565Leu) and FXIII codon 1704 A>G (p. Glu568=).

**Table 4:** Protective and detrimental mutations in control and patient subjects.

Protective	*p*-Value	Detrimental	*p*-Value
EPCR haplotypes A2/A2	0.006	Homozygous MTHFR C677T	0.04
Wild FXIII Val34Leu	0.001	Homozygous MTHFR A1298C	0.03
Heterozygous FXIII c. 1694 C>T p. Pro565Leu	0.03	Heterozygous FXIII Val34Leu	0.02
Homozygous FXIII c. 1694 C>T p. Pro565Leu	0.03	Wild FXIII c. 1694 C>T p. Pro565Leu	0.01

## Data Availability

All the data reported in this work are presented in the manuscript.
